# Early prophylactic heparin use is associated with reduced mortality in patients with non-traumatic subarachnoid hemorrhage

**DOI:** 10.3389/fneur.2026.1753639

**Published:** 2026-03-11

**Authors:** Haiyan Xiang, Xiujuan Chen, Laiyou Wang, Weihua Lai

**Affiliations:** 1 School of Medicine, South China University of Technology, Guangzhou, China; 2 Department of Clinical Pharmacy, Guangdong Provincial People’s Hospital (Guangdong Academy of Medical Sciences), Southern Medical University, Guangzhou, China; 3 Center of Medical Big Data, Guangdong Provincial People’s Hospital (Guangdong Academy of Medical Sciences), Southern Medical University, Guangzhou, China; 4 Guangdong Provincial Key Laboratory of Artificial Intelligence in Medical Image Analysis and Application, Guangzhou, China

**Keywords:** eICU-CRD, in-hospitalmortality, MIMICIV, non-traumatic subarachnoid hemorrhage, prophylactic heparin

## Abstract

**Background:**

Heparin may mitigate secondary brain injury in subarachnoid hemorrhage (SAH), but its effect on survival in non-traumatic SAH (NSAH) remains uncertain. This study aimed to investigate the association between early prophylactic heparin use (within 72 h of admission) and mortality outcomes in patients with NSAH.

**Methods:**

We performed a retrospective cohort study using the Medical Information Mart for Intensive Care (MIMIC) and the eICU Collaborative Research Database (eICU-CRD). Patients were stratified by early heparin use. Cox models, Kaplan–Meier (KM) curves, subgroup analyses, and comprehensive propensity score–based sensitivity analyses were applied to assess the robustness of the observed associations. The primary outcome was in-hospital mortality; 28-, 90-, 180-, and 365-day mortality were secondary outcomes. Findings were validated using the eICU-CRD cohort.

**Results:**

In the MIMIC-IV cohort, early heparin use was associated with lower in-hospital mortality (HR 0.62, 95% CI 0.39–0.97, *p* = 0.037), which was confirmed in the eICU-CRD cohort (HR 0.47, 95% CI 0.22–1.00, *p* = 0.049). Consistent reductions were also observed for 28-day (HR 0.57, *p* = 0.009), 90-day (HR 0.62, *p* = 0.010), 180-day (HR 0.63, *p* = 0.009), and 365-day mortality (HR 0.61, *p* = 0.004) in the MIMIC-IV cohort. Subgroup analyses and KM curves further supported these findings. Propensity score-based sensitivity analyses further validated the robustness of these findings in both cohorts.

**Conclusion:**

Early prophylactic heparin was associated with lower risk-adjusted short- and long-term mortality in NSAH.

## Introduction

NSAH is the third most common subtype of stroke (1). Despite its low incidence, representing only 5–10% of all strokes (2), NSAH is a critical condition with high mortality and disability rates caused by secondary brain injury such as cerebral vasospasm, neuroinflammation, and microthrombi (3, 4). However, effective treatments for secondary brain injury remain limited because of the multifactorial nature of the pathogenesis (4).

Heparin is a widely used anticoagulant, primarily preventing thrombosis by inhibiting thrombin generation through enhanced antithrombin III activity (5). Furthermore, the therapeutic potential of heparin extends beyond its anticoagulant properties. For example, it improves cerebral blood flow by suppressing endothelin-1 expression, enhances neuroprotective effects, and reduces inflammatory mediator release by inhibiting nuclear factor kappa B activation (6–9). Heparin provides multifaceted protection against secondary brain injury through various mechanisms (4, 10). Consequently, heparin may represent one of the most promising neuroprotective agents for SAH. Nevertheless, the clinical application of heparin in patients with SAH remains controversial, primarily due to concerns about the risk of intracranial rebleeding (11). This balance between potential neuroprotection and bleeding risk is the core of the clinical controversy surrounding heparin in SAH patients. Existing guidelines provide limited and inconclusive recommendations regarding heparin use in this population: heparin is generally not recommended prior to intracranial aneurysm treatment, whereas its use following aneurysm intervention may be considered on an individual basis (2, 12, 13).

In clinical practice, subcutaneous administration is the most common route for heparin prophylaxis, mainly owing to its favorable safety profile, predictable pharmacokinetic properties, and a lower risk of bleeding compared with intravenous infusion (14, 15). Moreover, subcutaneous heparin may be safer and better tolerated in patients with hemorrhagic stroke, given its more manageable hemorrhagic risk.

Compared with low molecular weight heparin, unfractionated heparin (UFH) demonstrates a greater binding affinity to endothelial cells, thereby exerting more potent anti-inflammatory effects, which are considered critical for mitigating neuroinflammation following SAH (15). Nonetheless, the role of early prophylactic administration of unfractionated heparin in patients with NSAH remains intriguing and warrants further investigation (4, 16–18).

However, there was still controversy about the association between early unfractionated heparin administration and mortality in NSAH patients. This study examines its early prophylactic administration (within 72 h of admission) on mortality in this population.

## Method

### Data source

We conducted a retrospective cohort study using data from the MIMIC-IV (version 3.1) database, a large, high-quality clinical dataset containing comprehensive information on patients admitted to the intensive care units (ICUs) of Beth Israel Deaconess Medical Center (BIDMC) between 2008 and 2022 (19). For external validation, we utilized the eICU-CRD cohort, which includes data from more than 200,000 ICU admissions across multiple hospitals in the United States (20).

Both databases were accessed in accordance with the approvals granted by the Institutional Review Boards of the Massachusetts Institute of Technology and BIDMC. The author, Haiyan Xiang, completed the Collaborative Institutional Training Initiative Program and obtained a passing score on the “Data or Sample Only Research” exam (Record ID: 60724732) on January 7, 2024, thereby gaining authorization to extract and analyze data from both databases.

Because all data were fully de-identified, the requirement for informed consent was waived by the BIDMC Ethics Committee. The study was conducted in accordance with the ethical principles outlined in the Declaration of Helsinki.

Patients diagnosed with NSAH were enrolled in this study according to the International Classification of Diseases, Ninth Revision (code: 430) and Tenth Revision (codes: I60, I600, I6001, I6002, I601, I6010, I6011, I6012, I602, I6020, I6021, I6022, I603, I6031, I6032, I604, I605, I6051, I6052, I606, I607, I608, I609) (Supplementary Table S1). The exclusion criteria were established according to the clinical rationale and data quality assurance protocols, including (1) minors (age <18 years); (2) not on their first admission; (3) length of stay (LOS) < 72 h; (4) administration of other anticoagulants during the initial 72-h period; (5) failure to transfer to intensive care unit (ICU) within 24 h of admission; (6) electronic medicine administration record documentation is missing; and (7) demographic clinical information is missing.

Because most severity scoring systems used in this study are derived from data collected within the first 24 h of ICU admission, we included only patients who were admitted to the ICU within 24 h of hospital admission. To ensure an adequate exposure window for heparin administration and to exclude early mortality cases, we excluded patients with a hospital LOS less than 72 h. Notably, patients with LOS < 72 h are less likely to receive heparin due to early death or extremely severe conditions, and their mortality is not associated with heparin use (Supplementary Table S2). This criterion ensured that all baseline severity assessments reflected the patients’ initial physiological state in a standardized manner.

### Sample size calculation

In this retrospective analysis, the sample size was determined by the total number of patients in the existing database. Thus, we estimated effect sizes through power analysis, employing PASS 2025 software.

### Exposure factors, covariates, and outcomes

The exposure variable was defined as subcutaneous heparin administration within the first 72 h after hospital admission. Medication exposure was confirmed using synthesized data from the electronic medicine administration record and pharmacy records to ensure the drugs were actually administered. Patients were divided into two groups: a heparin group and a non-heparin group, based on the administration of heparin via subcutaneous injection within 72 h of admission. We excluded patients who used any other anticoagulants. We analyzed the total heparin dose administered within 72 h of admission to investigate its relationship with mortality. The covariate variables extracted include:(1) demographic information: age, gender, and race;(2) clinical severity scores: Glasgow Coma Scale (GCS), Charlson Comorbidity Index (CCI), acute physiology score III (APS III), simplified acute physiology score II (SAPS II), sequential organ failure assessment (SOFA), and Oxford acute severity of illness score (OASIS);(3) vital signs: systolic blood pressure (SBP), diastolic blood pressure (DBP), mean blood pressure (MBP), respiratory rate, heart rate, peripheral oxygen saturation (SPO₂), and temperature;(4) laboratory parameters: hemoglobin, glucose, platelets, white blood cell (WBC) count, chloride, potassium, sodium, blood urea nitrogen (BUN), creatinine, bicarbonate, activated partial thromboplastin time (APTT), prothrombin time (PT), and international normalized ratio (INR);(5) comorbidities and personal history: hypertension, diabetes, stroke history, long-term anticoagulant use, cerebral edema, hydrocephalus, cerebral infarction, and epilepsy;(6) interventions within 72 h of admission: nimodipine administration, mechanical ventilation, vasopressor use, endovascular intervention, and open surgery.

The primary outcome was in-hospital mortality, and secondary outcomes included 28-day, 90-day, 180-day, and 365-day mortality. All statistical associations between early heparin exposure and clinical outcomes were evaluated using Cox proportional hazards regression models, ensuring methodological consistency across analyses.

### Statistical analysis

Continuous variables were summarized as mean ± standard deviation or median with interquartile range (IQR) and compared between groups using t-tests or Mann–Whitney U tests. Categorical variables were presented as frequencies (percentages) and assessed using chi-square or Fisher’s exact tests. In the MIMIC-IV cohort, we constructed a series of multivariable Cox regression models to assess the independent association between early heparin exposure and mortality outcomes (including in-hospital, 28-day, 90-day, 180-day, and 365-day mortality). Covariates for multivariable adjustment were systematically identified via two approaches: clinical plausibility assessment and the inclusion of variables with significant baseline differences. Subgroup analyses were stratified by gender, age (< 65 years; ≥ 65 years), PAASH grade, hypertension, and diabetes. KM survival curves were utilized to visualize the stability of hazard ratios in survival analysis.

To address potential baseline confounding between the heparin and non-heparin groups, a comprehensive propensity score–based sensitivity analysis framework was implemented after multiple imputation for missing values. All baseline covariates with differences and important clinical covariates were included to construct a Cox regression model for calculating propensity scores.

Multiple propensity score–based analytical approaches were subsequently applied to assess the robustness of the association, including propensity score adjustment, propensity score matching (PSM; 1:1 nearest-neighbor matching with a caliper width of 0.2), and several weighting methods, namely inverse probability of treatment weighting (IPTW), standardized mortality ratio weighting (SMRW), propensity adjustment weighting (PA), and overlap weighting (OW). Cox proportional hazards regression models were employed to analyze the association between heparin use and in-hospital mortality across models.

The eICU-CRD was used for external validation to assess the robustness and generalizability of findings. Due to the absence of follow-up data in eICU-CRD, only in-hospital mortality could be evaluated. The same variable definitions, exclusion criteria, and multivariable Cox regression modeling strategies used in the MIMIC-IV cohort were applied to the eICU-CRD cohort to ensure methodological consistency. The aforementioned propensity score-based sensitivity analyses were also performed in the eICU-CRD cohort to maintain methodological uniformity between the two cohorts.

Missing values in covariates were addressed using a multivariate single imputation method. This approach utilized an iterative imputer, with a Bayesian ridge model serving as the estimator in each step of the round-robin imputation process. Statistical significance was defined as a *p*-value < 0.05. The R software (version 4.3.3) and Free Statistics software (version 2.2) were used for all statistical studies.

## Results

### Patient characteristics

**Figure 1:**
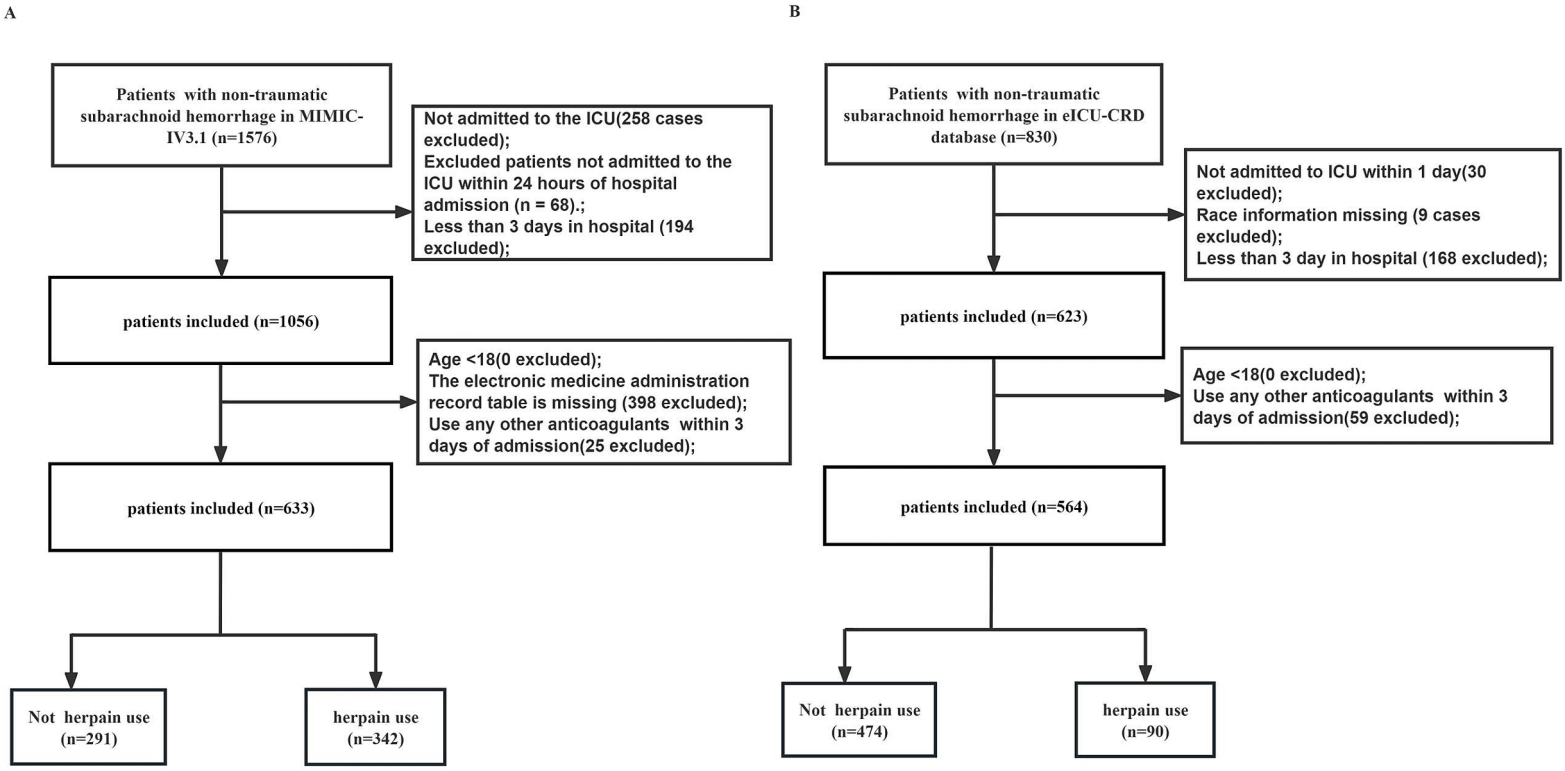
Flow chart of patient selection of the MIMIC-IV cohort **(A)** and the eICU-CRD cohort **(B)**.

A total of 633 patients with non-traumatic subarachnoid hemorrhage (NSAH) were included from the MIMIC-IV cohort, with 342 in the heparin group and 291 in the non-heparin group (Figure 1). Among all patients, 365 (57.7%) were female, and the median age was 62 years (IQR 52–74). The majority of patients were White (56.6%). The median GCS score was 13 (IQR 7–15), suggesting a relatively preserved level of consciousness. Baseline characteristics were generally comparable between the heparin and non-heparin groups (Table 1). However, the heparin group had a significantly higher GCS score and lower severity scores, including SAPII, APS III, and OASIS (all *p* < 0.05; Table 1). Regarding early interventions within the first 72 h after admission, significant differences were observed between the two groups: the heparin group had a higher proportion of patients receiving nimodipine (47.7% vs. 29.9%, *p* < 0.001), while the non-heparin group had higher rates of mechanical ventilation (45.4% vs. 32.5%, *p* = 0.001) and open surgery (9.6% vs. 4.4%, *p* = 0.014). The number of patients with APTT values exceeding 2.5 times the standard limit was similar between the heparin and control groups in both 2 cohorts (Table 1; Supplementary Table S3). The validation cohort from the eICU-CRD included 564 patients, of whom 90 (16.0%) were in the heparin group. Baseline characteristics were generally similar to those in the MIMIC-IV cohort (Supplementary Table S3).

**Table 1:** Characteristics of the included population in the MIMIC-IV cohort.

Variables	Overall	Non heparin	Heparin	*p* value
	*N*^t^ = 633	*N* = 291	*N* = 342	
Age, Mean ± SD^a^	62.56 (51.60, 73.56)	61.35 (50.97, 72.27)	63.44 (51.98, 73.58)	0.337
Gender, *n* (%)				
Female	365 (57.7)	160 (55.0)	205 (59.9)	0.239
Male	268 (42.3)	131 (45.0)	137 (40.1)	0.239
Race, *n* (%)				
White	358 (56.6)	168 (57.7)	190 (55.6)	0.464
Asian	25 (3.9)	14 (4.8)	11 (3.2)	0.464
Black	43 (6.8)	16 (5.5)	27 (7.9)	0.464
Other	207 (32.7)	93 (32.0)	114 (33.3)	0.464
Clinical scores				
GCS^b^(score), Median (IQR^c^)	13.00 (7.00, 15.00)	12.00 (6.00, 15.00)	14.00 (8.75, 15.00)	<0.001
PAASH^d^ Scale, *n* (%)				
I	227 (36.0)	85 (29.3)	142 (41.8)	0.001
II	144 (22.9)	64 (22.1)	80 (23.5)	0.001
III	100 (15.9)	47 (16.2)	53 (15.6)	0.001
IV	96 (15.2)	56 (19.3)	40 (11.8)	0.001
V	63 (10.0)	38 (13.1)	25 (7.4)	0.001
CCI ^e^ (score), Median (IQR)	4.00 (2.00, 6.00)	4.00 (2.00, 6.00)	4.00 (2.00, 6.00)	0.599
SAPS II^f^ (score), Median (IQR)	30.00 (24.00, 39.00)	32.00 (25.00, 40.00)	29.00 (22.25, 37.00)	0.005
APS III^g^ (score), Median (IQR)	31.00 (24.00, 43.00)	33.00 (25.00, 46.00)	31.00 (23.25, 42.00)	0.033
SOFA^h^ (score), Median (IQR)	0.00 (0.00, 1.00)	0.00 (0.00, 1.00)	0.00 (0.00, 1.00)	0.158
OASIS^i^ (score), Median (IQR)	29.00 (24.00, 35.75)	31.00 (26.00, 37.00)	28.00 (23.00, 35.00)	<0.001
Comorbidities and personal history, *n* (%)				
Stroke history	36 (5.7)	12 (4.1)	24 (7.0)	0.163
Long-term anticoagulants	56 (8.8)	27 (9.3)	29 (8.5)	0.832
Hypertension	346 (54.7)	152 (52.2)	194 (56.7)	0.293
Diabetes	95 (15.0)	44 (15.1)	51 (14.9)	1
Cerebral edema	171 (27.0)	79 (27.1)	92 (26.9)	1
Hydrocephalus	198 (31.3)	112 (38.5)	86 (25.1)	<0.001
Cerebral infarction	103 (16.3)	47 (16.2)	56 (16.4)	1
Epilepsy	37 (5.8)	20 (6.9)	17 (5.0)	0.397
Vital signs				
SBP^j^ (mmHg), Median (IQR)	132.00 (116.50, 145.00)	130.00 (115.00, 144.00)	133.00 (119.00, 147.00)	0.143
DBP^k^ (mmHg), Median (IQR)	71.00 (62.00, 81.00)	71.00 (62.00, 80.00)	72.00 (62.00, 82.00)	0.675
MBP^l^ (mmHg), Median (IQR)	91.33 (82.17, 101.33)	90.67 (82.42, 99.33)	92.33 (81.67, 102.33)	0.233
Respiratory rate (breaths/min), Median (IQR)	17.50 (15.00, 20.00)	17.00 (15.00, 20.00)	18.00 (15.00, 20.00)	0.819
Heart rate (beats/min), Median (IQR)	84.00 (70.00, 113.50)	84.00 (69.25, 110.00)	84.00 (71.00, 120.00)	0.499
SpO₂^m^ (%), Median (IQR)	98.00 (96.00, 100.00)	98.00 (96.00, 100.00)	98.00 (96.00, 100.00)	0.143
Temperature (°C), Median (IQR)	36.89 (36.56, 37.11)	36.83 (36.56, 37.11)	36.89 (36.61, 37.11)	0.549
Laboratory parameters				
Hemoglobin (g/dL), Median (IQR)	12.20 (10.93, 13.47)	12.30 (10.80, 13.50)	12.20 (11.00, 13.40)	0.876
Glucose (mg/dL), Median (IQR)	126.00 (105.00, 154.00)	132.00 (107.00, 166.00)	123.00 (105.00, 148.00)	0.013
Platelets (10^9^/L), Median (IQR)	215.00 (171.75, 259.00)	210.00 (167.00, 259.75)	217.00 (176.25, 256.00)	0.356
WBC^n^ (10^9^/L), Median (IQR)	11.20 (8.80, 14.03)	11.70 (9.10, 14.88)	10.80 (8.62, 13.30)	0.028
Chloride (mmol/L), Median (IQR)	104.00 (101.00, 107.00)	104.00 (101.00, 107.00)	104.00 (102.00, 107.00)	0.498
Potassium (mmol/L), Median (IQR)	3.90 (3.60, 4.30)	3.90 (3.55, 4.30)	3.90 (3.60, 4.20)	0.643
Sodium (mmol/L), Median (IQR)	139.00 (137.00, 141.00)	139.00 (137.00, 141.00)	139.00 (137.00, 141.00)	0.437
BUN^o^ (mg/dL), Median (IQR)	14.00 (10.00, 18.00)	14.00 (11.00, 19.00)	13.00 (10.00, 17.25)	0.048
Creatinine (mg/dL), Median (IQR)	0.80 (0.60, 1.00)	0.80 (0.60, 1.00)	0.80 (0.60, 0.90)	0.256
Bicarbonate (mmol/L) Median (IQR)	22.00 (20.00, 24.00)	22.00 (20.00, 24.00)	22.00 (20.00, 24.00)	0.806
APTT^p^ (s), Median (IQR)	28.00 (25.80, 30.83)	28.00 (26.00, 30.75)	28.00 (25.72, 31.08)	0.775
PT (s)^q^, Median (IQR)	12.20 (11.50, 13.00)	12.10 (11.40, 12.93)	12.30 (11.50, 13.10)	0.251
INR^r^, Median (IQR)	1.10 (1.10, 1.20)	1.10 (1.08, 1.20)	1.10 (1.10, 1.20)	0.169
Interventions within 72 h of admission				
Nimodipine	250 (39.5)	87 (29.9)	163 (47.7)	<0.001
Mechanical ventilation	243 (38.4)	132 (45.4)	111 (32.5)	0.001
Vasopressors	84 (13.3)	39 (13.4)	45 (13.2)	1
Endovascular intervention	193 (30.5)	77 (26.5)	116 (33.9)	0.052
Open surgery	43 (6.8)	28 (9.6)	15 (4.4)	0.014
Clinical outcomes				
APTT > 87.5	60 (9.5)	30 (10.3)	30 (8.8)	0.645
Los^s^ hospital	12.97 (7.41, 21.90)	13.86 (7.59, 24.92)	12.34 (7.35, 19.62)	0.095
Death in hosp	89 (14.1)	54 (18.6)	35 (10.2)	0.004
Death within 28 days	97 (15.3)	59 (20.3)	38 (11.1)	0.002
Death within 90 days	126 (19.9)	73 (25.1)	53 (15.5)	0.004
Death within 180 days	139 (22.0)	80 (27.5)	59 (17.3)	0.003
Death within 365 days	152 (24.0)	88 (30.2)	64 (18.7)	0.001

^a^SD, standard deviation; ^b^IQR, interquartile range; ^c^LOS, length of stay; ^d^GCS, glasgow coma scale; ^e^PAASH, prognosis on admission of aneurysmal subarachnoid hemorrhage; ^f^CCI, charlson comorbidity index; ^g^SAPS II, simplified acute physiology score II; ^h^APS III, acute physiology score III; ^i^SOFA, sequential organ failure assessment; ^j^OASIS, oxford acute severity of illness score; ^k^SBP, systolic blood pressure; ^l^DBP, diastolic blood pressure; ^m^MBP, mean blood pressure; ^n^SpO₂, percutaneous oxygen saturation; ^o^WBC, white blood cell; ^p^BUN, blood urea nitrogen; ^q^APTT, activated partial thromboplastin time; ^r^PT, prothrombin time; ^s^INR, international normalized ratio; ^t^N, number.

The missing rate of each variable is shown in Supplementary Table S4. A power analysis yielded a power value of 0.82 (Supplementary Table S5), which exceeds the commonly accepted threshold of 0.80. This suggests that the study has sufficient statistical power to detect meaningful differences. The validation cohort from eICU-CRD demonstrated a slightly lower power of 0.79, which, although marginally below 0.80, was still considered acceptable for exploratory external validation (Supplementary Table S6).

### Primary outcome

Compared with the non-heparin group, the heparin group exhibited consistently lower mortality across all time points in the MIMIC-IV cohort, including in-hospital (10.2% vs. 18.6%, *p* = 0.004), 28-day (11.1% vs. 20.3%, *p* = 0.002), 90-day (15.5% vs. 25.1%, *p* = 0.004), 180-day (17.3% vs. 27.5%, *p* = 0.003), and 365-day mortality (18.7% vs. 30.2%, *p* = 0.001; Table 1).

Multivariable Cox proportional hazards regression analyses confirmed that early prophylactic heparin use was independently associated with a reduced risk of mortality across all models (Table 2). Specifically, In the fully adjusted model (Model 4), the heparin group had significantly lower risks of in-hospital mortality (HR 0.62, 95% CI 0.39–0.97, *p* = 0.037), 28-day mortality (HR 0.57, 95% CI 0.37–0.87, *p* = 0.009), 90-day mortality (HR 0.62, 95% CI 0.42–0.89, *p* = 0.010), 180-day mortality (HR 0.63, 95% CI 0.44–0.89, *p* = 0.009), and 365-day mortality (HR 0.61, 95% CI 0.44–0.86, *p* = 0.004). These associations remained stable across sensitivity models with varying degrees of covariate adjustment.

**Table 2:** Cox proportional hazards regression model results of early heparin administration and mortality outcomes.

Outcome	Univariate^a^		Model 1^b^	*p* value	Model 2^c^		Model 3^d^		Model 4^e^	
	HR^f^ (95% CI^g^)	*p* value	HR (95% CI)		HR (95% CI)	*p* value	HR (95% CI)	*p* value	HR (95% CI)	*p* value
Death within 28 days (MIMIC-IV)	0.52 (0.34–0.78)	0.001	0.50 (0.33–0.75)	<0.001	0.54 (0.36–0.83)	0.005	0.57 (0.37–0.89)	0.012	0.57 (0.37–0.87)	0.009
Death within 90 days (MIMI-IV)	0.57 (0.40–0.81)	0.002	0.55 (0.39–0.79)	<0.001	0.59 (0.41–0.86)	0.005	0.65 (0.44–0.94)	0.024	0.62 (0.42–0.89)	0.01
Death within 180 days (MIMIC-IV)	0.58 (0.41–0.81)	0.001	0.56 (0.40–0.78)	<0.001	0.61 (0.43–0.87)	0.006	0.65 (0.45–0.92)	0.017	0.63 (0.44–0.89)	0.009
Death within 365 days (MIMIC-IV)	0.57 (0.41–0.78)	<0.001	0.55 (0.40–0.75)	<0.001	0.60 (0.43–0.84)	0.003	0.62 (0.44–0.88)	0.007	0.61 (0.44–0.86)	0.004
In-hospital death (MIMIC-IV)	0.58 (0.38–0.90)	0.014	0.57 (0.37–0.87)	0.01	0.58 (0.37–0.91)	0.018	0.62 (0.39–0.98)	0.043	0.62 (0.39–0.97)	0.037
In-hospital death (eICU-CRD)	0.48 (0.24–0.95)	0.036	0.48 (0.24–0.97)	0.04	0.42 (0.20–0.87)	0.02	0.54 (0.26–1.10)	0.091	0.47 (0.22–1.00)	0.049

^a^No covariates adjusted. ^b^Model 1, Adjusted for age, gender, and race. ^c^Model 2, Adjusted for age, gender, race, GCS, PAASH, SAPSII, APSIII, OASIS, CCI, SOFA. ^d^Model 3, Adjusted for variables with *p* < 0.05 in Table 1, including GCS, PAASH, SAPSII, APSIII, OASIS, glucose, WBC, BUN, hydrocephalus, mechanical ventilation, nimodipine, and open surgery. ^e^Model 4, Adjusted for age, gender, race, GCS, PAASH, SAPSII, APSIII, OASIS, CCI, SOFA, long-term anticoagulants, stroke history, hypertension, diabetes, and nimodipine. ^f^hazard ratio. ^g^confidence interval.

To further assess the robustness of the observed association, we conducted a series of propensity score–based sensitivity analyses in both cohorts (Table 3). In the MIMIC-IV cohort (n = 633), a consistent inverse association between early prophylactic heparin use and in-hospital mortality was observed across multiple analytical approaches. Specifically, the unmatched crude model (HR = 0.58, 95% CI: 0.38–0.90, *p* = 0.014), the multivariable-adjusted model (HR = 0.58, 95% CI: 0.36–0.94, *p* = 0.027), the propensity score–matched model (HR = 0.57, 95% CI: 0.34–0.95, *p* = 0.030), and the IPTW-weighted model (HR = 0.59, 95% CI: 0.38–0.91, *p* = 0.028) all demonstrated statistically significant reductions in in-hospital mortality associated with heparin use.

**Table 3:** Sensitivity analyses of the association between early prophylactic heparin use and in-hospital mortality.

Analytical model	MIMI-IV cohort		eICU-CRD cohort	
	HR^a^ (95% CI ^j^)	*p* value	HR (95% CI)	*p* value
Unmatched. crude^b^	0.58 (0.38 ~ 0.9)	0.014	0.48 (0.24 ~ 0.95)	0.036
Multivariable. adjusted^c^	0.58 (0.36 ~ 0.94)	0.027	0.42 (0.19 ~ 0.93)	0.031
PropensityScore. adjuste^d^	0.68 (0.43 ~ 1.07)	0.098	0.46 (0.21 ~ 1)	0.049
PropensityScore. Matched^e^	0.57 (0.34 ~ 0.95)	0.03	0.47 (0.18 ~ 1.22)	0.123
Weighted. IPTW^f^	0.59 (0.38 ~ 0.91)	0.028	0.51 (0.24 ~ 1.07)	0.144
Weighted. SMRW^g^	0.62 (0.4 ~ 0.98)	0.075	0.42 (0.21 ~ 0.84)	0.026
Weighted. PA^h^	0.72 (0.43 ~ 1.2)	0.162	0.4 (0.16 ~ 0.99)	0.021
Weighted. Ow^i^	0.68 (0.35 ~ 1.31)	0.098	0.45 (0.16 ~ 1.26)	0.041

^a^Hazard ratio. ^b^Adjusted for GCS, PAASH, SAPSII, APSIII, OASIS, glucose, WBC, BUN, hydrocephalus, mechanical ventilation, nimodipine, open surgery, age, gender, race, GCS, PAASH, SAPSII, APSIII, OASIS, CCI, SOFA, long-term anticoagulants, stroke history, hypertension, diabetes, nimodipine, and vasopressors. ^d^Propensity score-adjusted model (adjusted for the same covariates as the multivariable adjusted model). ^e^Propensity score matching (1:1 nearest neighbor matching with a caliper of 0.2, adjusting for the same covariates as the multivariable adjusted model). ^f^Inverse probability of treatment weighting (weighted by the propensity score derived from the same covariate set as above). ^g^Standardized mortality ratio weighting (weighted by the propensity score derived from the same covariate set as above). ^h^Propensity adjustment weighting (weighted by the propensity score derived from the same covariate set as above). ^i^Overlap weighting (weighted by the propensity score derived from the same covariate set as above). ^j^Confidence interval.

Although the propensity score–adjusted model (HR = 0.68, *p* = 0.098) and the SMRW-weighted model (HR = 0.62, *p* = 0.075) did not reach conventional levels of statistical significance, the hazard ratios were consistently below 1, indicating a directionally consistent protective effect.

In the eICU-CRD validation cohort, the heparin group showed a consistent association with lower in-hospital mortality across all models. In the unadjusted analysis, the heparin group had a significantly lower risk of in-hospital death (HR 0.48, 95% CI 0.24–0.95, *p* = 0.036). After multivariable adjustment (Model 4), this association remained statistically significant (HR 0.47, 95% CI 0.22–1.00, *p* = 0.049). Although one intermediate model did not reach statistical significance, the direction of effect was consistent with the overall trend (Table 2). Furthermore, significant associations were also observed in the propensity score–adjusted model (HR = 0.46, 95% CI: 0.21–1.00, p = 0.049), the SMRW-weighted model (HR = 0.42, 95% CI: 0.21–0.84, *p* = 0.026), the propensity adjustment–weighted model (HR = 0.40, 95% CI: 0.16–0.99, *p* = 0.021), and the overlap weighting model (HR = 0.45, 95% CI: 0.16–1.26, *p* = 0.041).

Although the propensity score–matched model (HR = 0.47, *p* = 0.123) and the IPTW-weighted model (HR = 0.51, *p* = 0.144) did not achieve statistical significance, the estimated hazard ratios consistently favored heparin use, supporting the overall robustness of the protective association (Table 3).

A dose–response trend was further evaluated in the MIMIC-IV cohort. Increasing cumulative heparin dose within the first 72 h after admission was associated with progressively lower mortality across all endpoints (all *p* for trend < 0.01). Although not all individual dosage groups reached statistical significance, the overall pattern indicated a dose-dependent protective effect of early heparin use. In contrast, due to the limited number of patients receiving heparin in the eICU cohort, a dose–response analysis could not be performed (Table 4; Supplementary Table S7).

**Table 4:** Cox proportional hazards regression model results for heparin cumulative dose in the first 72 h and mortality outcomes in the MIMIC-IV cohort (adjusted for model 4).

Outcome	Variable	Dose level	Model 4^a^	
			HR^b^ CI^c^	*p* value
In-hospital death	Early heparin total dose (72 h)	0	1 (reference)	
		5,000–10,000 units	0.80 (0.46–1.39)	0.436
		15,000 units	0.48 (0.22–1.04)	0.062
		≥ 20,000 units	0.54 (0.24–1.18)	0.120
		*p* for trend		0.006
Death within 28 days	Early heparin total dose (72 h)	0	1 (reference)	
		5,000–10,000 units	0.65 (0.38–1.12)	0.119
		15,000 units	0.62 (0.33–1.18)	0.143
		≥ 20,000 units	0.41 (0.19–0.93)	0.032
		*p* for trend		<0.001
Death within 90 days	Early heparin total dose (72 h)	0	1 (reference)	
		5,000–10,000 units	0.63 (0.39–1.01)	0.056
		15,000 units	0.76 (0.44–1.29)	0.308
		≥ 20,000 units	0.50 (0.26–0.95)	0.034
		*p* for trend		0.002
Death within 180 days	Early heparin total dose (72 h)	0	1 (reference)	
		5,000–10,000 units	0.64 (0.40–1.01)	0.053
		15,000 units	0.77 (0.47–1.28)	0.318
		≥ 20,000 units	0.50 (0.27–0.94)	0.031
		*p* for trend		<0.001
Death within 365 days	Early heparin total dose (72 h)	0	1 (reference)	
		5,000–10,000 units	0.62 (0.40–0.96)	0.033
		15,000 units	0.73 (0.45–1.20)	0.214
		≥ 20,000 units	0.52 (0.29–0.93)	0.026
		*p* for trend		<0.001

^a^Model 4, Adjusted for age, gender, race, GCS, PAASH, SAPSII, APSIII, OASIS, CCI, SOFA, long-term anticoagulants, stroke history, hypertension, diabetes, and nimodipine. ^b^hazard ratio; ^c^confidence interval.

**Figure 2:**
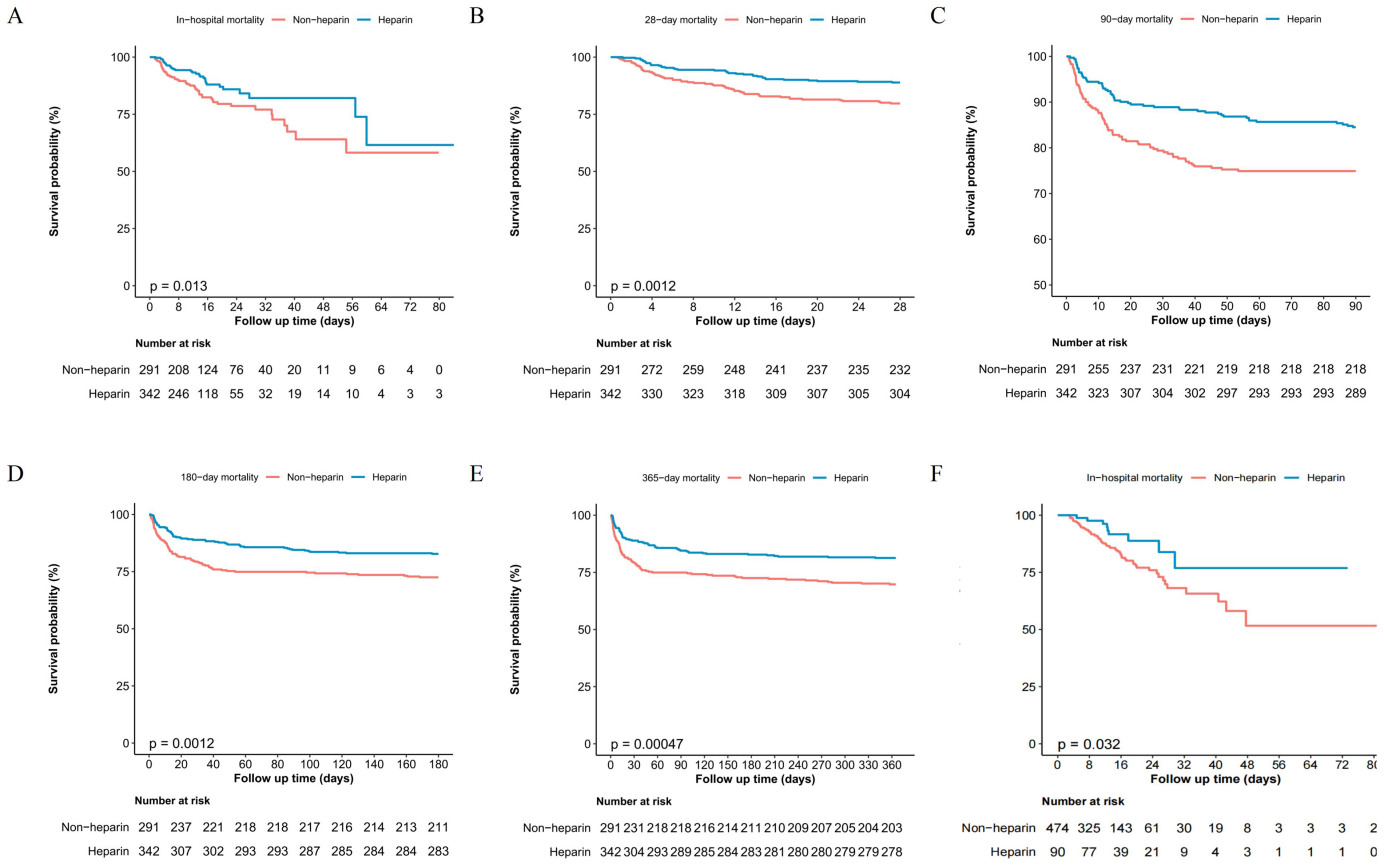
Kaplan–Meier survival analysis: **(A)** In-hospital mortality (MIMIC-IV cohort), **(B)** 28-day mortality (MIMIC-IV cohort), **(C)** 90-day mortality (MIMIC-IV cohort), **(D)** 180-day mortality (MIMIC-IV cohort), **(E)** 365-day mortality (MIMIC-IV), **(F)** In-hospital mortality (eICU-CRD cohort).

KM survival curves demonstrated a higher probability of in-hospital survival in the heparin group in both the MIMIC-IV and eICU cohorts. Consistently, in the MIMIC-IV cohort, patients receiving heparin also showed better survival at 28-day, 90-day, 180-day, and 365-day follow-ups (Figure 2).

**Figure 3:**
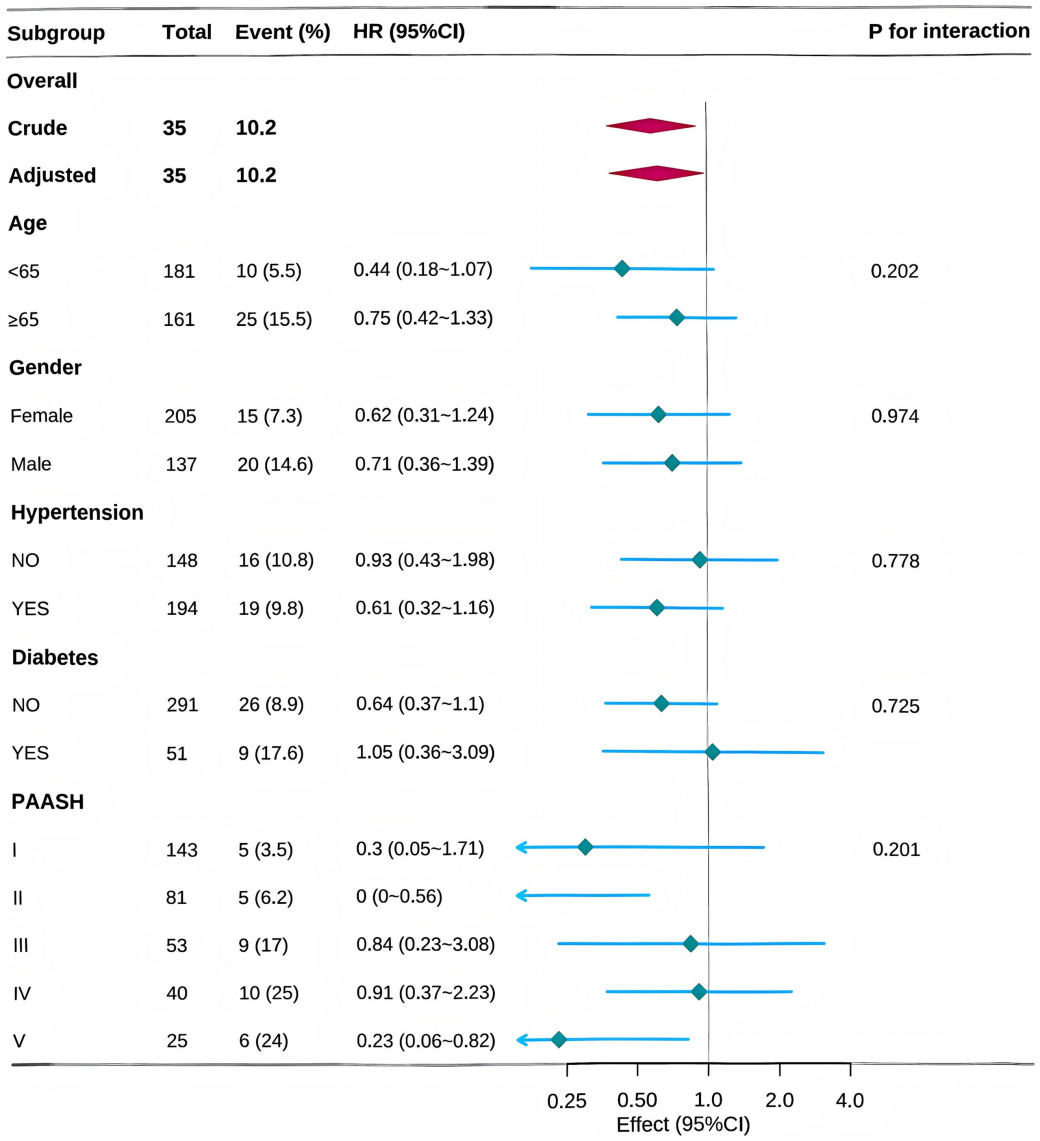
Subgroup analyses for in-hospital mortality in the MIMIC-IV cohort.

Subgroup analyses were performed to evaluate differences in the effect of heparin administration across various populations, stratified by gender, age (<65 years vs. ≥ 65 years), PAASH grade, hypertension, and diabetes, after adjusting for covariates included in Model 4 of Cox regression. Heparin administration showed a consistent association with in-hospital mortality across all subgroups. None of the stratification factors significantly modified this association (Figure 3; Supplementary Figure S1).

## Discussion

### Statement of key findings

Our study revealed that early heparin administration within 72 h of admission was associated with a reduced in-hospital mortality (HR = 0.62) and demonstrated a dose-dependent effect among NSAH patients. Patients in the non-heparin group had higher baseline severity scores (SAPS II, APS III, OASIS) and more frequently received mechanical ventilation and open surgery, which likely reflects clinical practice where clinicians tend to reserve heparin for patients with relatively milder initial presentations. However, even after adjusting for these baseline severity indices and early treatment differences in multivariable Cox regression analysis, the association between early heparin use and reduced mortality remained statistically significant. In the eICU-CRD validation cohort, the heparin group showed a consistent association with lower in-hospital mortality across all models. In the unadjusted analysis, the heparin group showed a significantly lower in-hospital death risk (HR 0.48, 95% CI 0.24–0.95, *p* = 0.036), and this association persisted statistically significantly after multivariable adjustment (Model 4, HR 0.47, 95% CI 0.22–1.00, *p* = 0.049). Although one intermediate model did not reach statistical significance, the direction of effect was consistent with the overall trend. The associations for other mortality time points remained consistent with the findings in all models. In NSAH patients, heparin administration was significantly associated with reductions in both short- and long-term mortality. The propensity score–based sensitivity analyses further supported the robustness of this association: despite partial inter-model deviations (attributed to model adaptability differences, residual confounding, and limited sample size in the eICU-CRD cohort), all models showed a consistent protective trend (HR values < 1) in both cohorts, and the core models (unmatched crude model, multivariable adjusted model) were consistently significant.

A dose–response trend was evaluated in the MIMIC-IV cohort. Although not all individual dosage groups showed statistically significant reductions compared to the reference group, a significant trend toward reduced all-cause mortality (referring to all death outcomes) with increasing heparin dosage was observed. This discrepancy may be explained by the relatively small sample sizes in some of the dosage groups, which could limit the ability to detect significant differences between groups. However, due to the limited number of patients receiving heparin in the eICU cohort, a dose–response analysis could not be performed in the eICU-CRD cohort.

These results support that early subcutaneous administration of heparin may confer protective benefits in patients with NSAH.

### Interpretation

Previous studies have not clearly established whether early administration of heparin can reduce mortality among patients with spontaneous SAH. One prior study reported that the use of low-molecular-weight heparin did not significantly decrease mortality, although it was associated with improved functional outcomes (16). However, that study included only 370 patients, and the limited sample size may have restricted its statistical power. Therefore, evidence regarding the effect of early heparin use on mortality in SAH remains insufficient. Our findings provide new evidence to fill this gap, demonstrating that early prophylactic heparin administration within 72 h of admission was significantly associated with lower short- and long-term mortality among NSAH patients.

Despite widespread clinical concerns regarding rebleeding risks associated with perioperative heparin administration (7), our findings further support that early prophylactic heparin administration is significantly associated with short- and long-term mortality reduction. This observation suggests that the primary physiological function of heparin may extend beyond its anticoagulant properties (21).

Consistent with our results, previous studies demonstrated that early heparin administration after undergoing aneurysm repair improved outcomes. A study by Hantsche et al. demonstrated that initiating prophylactic anticoagulation within 48 h after surgery significantly improved functional outcomes at 12 months, as measured by the glasgow outcome scale (OR = 0.422, *p* = 0.006). Moreover, the risk of systemic ischemia increased by 1.3% for each hour of delayed initiation of heparin therapy (OR = 1.013 per hour, 95% CI: 1.001–1.024). In contrast, patients who received anticoagulation within 24 h had a significantly lower incidence of systemic ischemia (OR = 0.259, *p* = 0.020) (16). Notably, heparin did not increase the risk of rebleeding (5.7% vs. 3.2% in the non-anticoagulated group, *p* = 0.958). Another retrospective cohort study showed that heparin administration within 48 h postoperatively significantly reduced the incidence of severe cerebral vasospasm and tended to have a more favorable outcome (4). There was no significant difference in rebleeding rates between the two groups [3.0% (6 cases) in the heparin group vs. 4.6% (9 cases) in the control group] (4). Animal studies further support that low-dose heparin significantly improves early outcomes without increasing the risk of rebleeding (22). These findings suggest that early heparin intervention may improve neurological outcomes without significantly increasing rebleeding risk.

The clinical application of heparin in NSAH patients remains largely empirical, with no standardized protocols for dosage or administration routes. Regarding administration routes, controversy persists between subcutaneous injection and intravenous infusion: some neurocritical care guidelines recommend initiating subcutaneous UFH 24 h after aneurysm embolization (14), but subcutaneous UFH is limited by low bioavailability, which may lead to inconsistent therapeutic effects. In contrast, intravenous infusion is considered a more reliable option for achieving stable blood concentrations, and an ongoing randomized controlled trial is currently investigating its efficacy and safety in aneurysmal SAH (18).

For heparin dosage, the Maryland Low-Dose Intravenous Heparin Infusion Protocol provides a practical perioperative reference: starting at 8 units per kilogram per hour (U/kg/h) (no loading dose) at 12 h post craniotomy, titrating up to 9 U/kg/h and 10 U/kg/h at 12 h intervals, and maintaining infusion for 12–16 days post ictus on the premise of confirmed aneurysm obliteration (16). Treatment duration also appears critical, as a meta-analysis reported that clinical benefits of heparin in NSAH patients are generally observed only when therapy exceeds 48 h (14). Regrettably, due to database limitations, we were unable to examine daily heparin dosage or exact treatment duration. However, this finding is highly consistent with our observation of a dose-dependent protective effect of heparin initiated within 72 h of admission, suggesting that sufficient subcutaneous heparin exposure duration and cumulative dose may be necessary to achieve meaningful reductions in in-hospital mortality among NSAH patients. Heparin may reduce mortality in patients with early NSAH through multiple synergistic mechanisms. Its primary mechanism involves inhibiting the secondary brain injury cascade (21). Inflammation, a key pathogenic factor in cerebral injury following SAH, is closely associated with the development of vasospasm and increased mortality (23–25). Due to its extremely high negative charge density, heparin interacts with various inflammatory mediators and immune proteins, exerting broad-spectrum anti-inflammatory effects (5, 21). It achieves these effects through several mechanisms: firstly, it inhibits the inhibiting nuclear factor kappa B signaling pathway and reduces pro-inflammatory gene expression; secondly, it binds to and neutralizes inflammatory cytokines while suppressing immune cell activation, thereby attenuating the microglia-driven inflammatory storm; thirdly, it increases the expression of the anti-inflammatory cytokine interleukin-10; finally, it downregulates adhesion molecule expression on the surface of endothelial cells (15, 23, 26–30). Cerebral vasospasm is closely associated with cerebral infarction and delayed cerebral ischemia (DCI) (1, 31), both of which are severe complications of SAH. Heparin counteracts cerebral vasospasm through multiple mechanisms of action (32, 33). For example, heparin enhances cerebral perfusion by neutralizing free radicals released from oxyhemoglobin, owing to its strong electronegative properties, and reduces vasospasm by antagonizing endothelin-1 (3, 6, 9, 10, 21, 34). In addition to being anti-inflammatory and anti-vasospasm, heparin reduces DCI through anticoagulation. Heparin inhibits factor Xa and thrombin by activating antithrombin III, thus reducing the risk of microthrombosis and DCI. This mechanism is consistent with the significant reduction in microvascular embolism observed in autopsy studies (4, 9, 10). Besides, Heparin preserves tight junction integrity and reduces blood–brain barrier permeability by inhibiting Matrix Metalloproteinase-9 activity, potentially decreasing cerebral edema volume, lowering intracranial pressure, and improving neurological outcomes (9, 30). This multimodal intervention, from the molecular to the systemic level, ultimately reduces the relative risk of combined mortality (21). Based on these potential mechanisms, heparin may provide a broad-spectrum protective effect against early brain injury in SAH and may contribute to improved long-term outcomes (11, 13, 15).

Although heparin may theoretically increase the risk of rebleeding, our preliminary APTT monitoring data showed that the proportion of patients with excessive APTT prolongation (defined as >2.5 times the upper normal limit) was comparable between the heparin and non-heparin groups across both cohorts, with no significant intergroup difference. This finding is consistent with previous reports that did not observe a significant increase in the risk of rebleeding with heparin administration (4, 17, 21). However, due to the limited sample size in this subgroup, these findings should be interpreted with caution.

### Strengths and weaknesses

Our study provides novel evidence that early prophylactic heparin administration within 72 h of admission is associated with reduced all-cause mortality in NSAH patients, a finding that was further supported by consistent results in the multicenter eICU-CRD validation cohort. These findings may offer valuable insights into the early management of NSAH. However, our study has some limitations. First, the retrospective design may introduce inherent biases that cannot be fully controlled. Even with comprehensive propensity score-related sensitivity analyses to balance baseline confounders, residual or unadjusted confounders may still exist, as reflected in the partial inter-model deviations observed in sensitivity analyses. These variations likely reflect unrecorded clinical decision factors that are inherent to retrospective studies. Second, the precise temporal sequence of certain neurological complications (such as hydrocephalus and cerebral edema) relative to heparin initiation could not be fully determined. Although these conditions were treated as baseline covariates and rigorously adjusted for in multivariable and propensity score–based analyses, potential misclassification of their timing cannot be entirely excluded. This limitation may introduce residual confounding and warrants cautious interpretation of the findings. Third, incomplete information regarding surgical procedures, specific SAH types, and detailed severity classifications or scores (e.g., Hunt–Hess grade) in both databases may have limited the precision of our analysis. For example, surgical timestamps were recorded only by day, without specific start and end times, preventing precise assessment of the temporal sequence between heparin administration and surgery. In addition, discontinuities in individual heparin administration records made it challenging to define fixed daily doses or standardized treatment durations, limiting the comprehensiveness of dose–response analyses. Nonetheless, we extracted all available clinical variables and severity assessments to mitigate this limitation as much as possible. Finally, we applied strict exclusion criteria to ensure methodological rigor, which inevitably reduced the sample size in certain subgroups, although the overall cohort remained adequate for statistical analysis. In addition, the number of heparin-treated patients in the eICU-CRD validation cohort was inherently small, which prevented further dose–response analyses.

Overall, these findings should be interpreted with caution, and larger, prospective randomized controlled trials are warranted to confirm our results, clarify optimal dosing strategies, and explore the underlying mechanisms of heparin in NSAH.

## Conclusion

In conclusion, our study demonstrates that early prophylactic administration of heparin is significantly associated with reduced short- and long-term mortality in patients with NSAH. Although the limited sample size precluded detailed subgroup analysis of coagulation parameters, APTT monitoring did not indicate a clear increase in excessive prolongation in the heparin group compared to controls. This may suggest that early prophylactic heparin administration, under careful monitoring, could be a safe and potentially beneficial strategy in selected patients.

## Data Availability

Publicly available datasets were analyzed in this study. This data can be found at: https://physionet.org/content/mimiciv/3.1/; https://physionet.org/content/eicu-crd-demo/2.0.1/.

## Glossary

SAH: Subarachnoid Hemorrhage
NSAH: Non-traumatic SAH
BIDMC: Beth Israel Deaconess Medical Center
GCS: Glasgow Coma Scale
PAASH: Prognosis on Admission of Aneurysmal Subarachnoid Hemorrhage
CCI: Charlson Comorbidity Index
SAPS II: Simplified Acute Physiology Score II
APS III: Acute Physiology Score III
SOFA: Sequential Organ Failure Assessment
OASIS: Oxford Acute Severity of Illness Score
MIMIC-IV: The Medical Information Mart for Intensive Care IV
eICU-CRD: eICU Collaborative Research Database
ICU: Intensive Care Unit
APTT: Activated Partial Thromboplastin Time
PT: prothrombin time
INR: international normalized ratio
PSM: Propensity Score Matching
OR: Odds Ratio
HR: Hazard Ratio
CI: Confidence Interval
DCI: Delayed Cerebral Ischemia
eMAR: Electronic Medication Administration Record
SBP: Systolic Blood Pressure
DBP: Diastolic Blood Pressure
MBP: Mean Blood Pressure
SPO₂: Percutaneous Oxygen SaturationWBC White Blood Cell
BUN: Blood Urea Nitrogen
PT: Prothrombin Time
INR: International Normalized Ratio
IQR: Interquartile Range
SD: Standard Deviation
KM: Kaplan–Meier
LOS: length of stay
UFH: Unfractionated Heparin
IPTW: Inverse Probability of Treatment Weighting
SMRW: Standardized Mortality Ratio Weighting
PA: Propensity Adjustment Weighting
OW: Overlap Weighting
U/kg/h: Units per Kilogram per hour
