# Supplementary material for: Early prophylactic heparin use is associated with reduced mortality in patients with non-traumatic subarachnoid hemorrhage
Source: Front Neurol. 2026 Mar 11;17:1753639. doi: 10.3389/fneur.2026.1753639 (PMC13012946; doi:10.3389/fneur.2026.1753639)
Supplement: Supplementary file 1 [file Data_Sheet_1.docx]

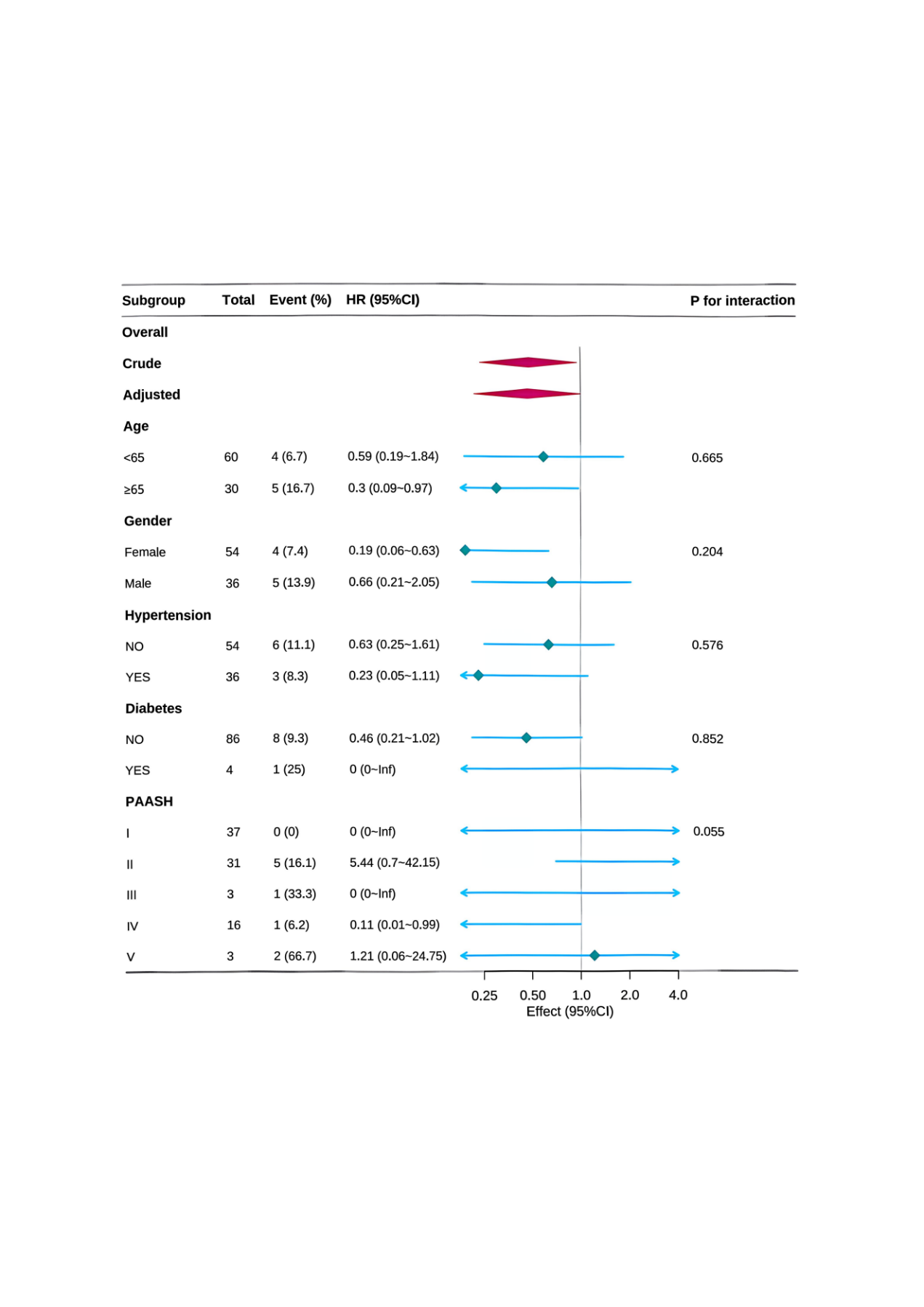


Supplemental Figure S1. Subgroup analyses for in-hospital mortality in the eICU-CRD cohort.

Table S1. Distribution of ICD codes for non-traumatic subarachnoid hemorrhage in the study population of the MIMIC-IV cohort

| ICD CODE^a^ | Count |
| --- | --- |
| I609 | 234 |
| 430 | 91 |
| I608 | 73 |
| I602 | 43 |
| I606 | 33 |
| I6031 | 31 |
| I6012 | 29 |
| I6011 | 22 |
| I6032 | 22 |
| I604 | 22 |
| I607 | 15 |
| I6001 | 6 |
| I6051 | 5 |
| I6052 | 5 |
| I6022 | 4 |
| I6002 | 3 |
| I6020 | 2 |
| I6010 | 1 |
| I6021 | 1 |

a: International Classification of Diseases; Some patients were assigned more than one ICD-10 code for non-traumatic subarachnoid hemorrhage during the same hospitalization. Each patient was counted only once for cohort inclusion, while all recorded ICD codes were retained for descriptive purposes in this table.

Table S2. Comparison of mortality outcomes between heparin and non-heparin groups in patients with hospital length of stay <72 hours and ≥72 hours.

| Outcome | Los^a^<72h Overall Mortality (%) | Los>=72h Mortality (%) | *P* value | Non-heparin Mortality (%) | Heparin Mortality (%) | *P* value |
| --- | --- | --- | --- | --- | --- | --- |
| In-hospital (MIMIC-IV) | 54(56.80) | 89 (14.1) | <0.001 | 51 (60.0) | 3 (30.0) | 0.095 |
| 28-day (MIMIC-IV) | 56(58.90) | 97 (15.3) | <0.001 | 53 (62.4) | 3 (30.0) | 0.086 |
| 90-day (MIMIC-IV) | 57(60.0) | 126 (19.9) | <0.001 | 54 (63.5) | 3 (30.0) | 0.083 |
| 180-day (MIMIC-IV) | 57(60.0) | 139 (22.0) | <0.001 | 54 (63.5) | 3 (30.0) | 0.083 |
| 365-day (MIMIC-IV) | 57(60.0) | 152 (24.0) | <0.001 | 54(63.5) | 3 (30.0) | 0.083 |
| In-hospital (eICU-CRD) | 64(41.0) | 84 (14.9) | <0.001 | 62(41.1) | 2(40.0) | 1.000 |

a: length of stay.

**Table S3.** Characteristics of the included population in the eICU-CRD cohort

| Variables | Overall | Non heparin | Heparin | *P* value |
| --- | --- | --- | --- | --- |
|  | N^t^=564 | N=474 | N=90 |  |
| Age, Mean ± SD^a^ | 58.0 (48.8, 70.0) | 58.0 (48.0, 71.0) | 59.0 (53.0, 68.0) | 0.529 |
| Gender, n (%) |  |  |  | 0.568 |
| Female | 323 (57.3) | 269 (56.8) | 54 (60) |  |
| Male | 241 (42.7) | 205 (43.2) | 36 (40) |  |
| Race, n (%) |  |  |  | 0.087 |
| White | 405 (71.8) | 348 (73.4) | 57 (63.3) |  |
| Asian | 14 ( 2.5) | 12 (2.5) | 2 (2.2) |  |
| Black | 58 (10.3) | 49 (10.3) | 9 (10) |  |
| Other | 87 (15.4) | 65 (13.7) | 22 (24.4) |  |
| Clinical scores |  |  |  |  |
| GCS ^b^(score), Median (IQR^c^) | 14.0 (10.0, 15.0) | 14.0 (9.8, 15.0) | 14.0 (11.0, 15.0) | 0.912 |
| PAASH^d^ Scale, n (%) |  |  |  | 0.033 |
| I | 223 (48.0) | 187 (48.7) | 36 (44.4) |  |
| II | 113 (24.3) | 86 (22.4) | 27 (33.3) |  |
| III | 37 ( 8.0) | 35 (9.1) | 2 (2.5) |  |
| IV | 63 (13.5) | 49 (12.8) | 14 (17.3) |  |
| V | 29 ( 6.2) | 27 (7) | 2 (2.5) |  |
| CCI ^e^ (score), Median (IQR) | 0.0 (0.0, 1.0) | 0.0 (0.0, 1.0) | 0.0 (0.0, 1.0) | 0.843 |
| SAPSⅡ^f^ (score), Median (IQR) | 23.0 (15.0, 32.2) | 24.0 (15.0, 32.0) | 21.5 (15.0, 33.0) | 0.787 |
| APS Ⅲ^g^ (score), Median (IQR) | 33.0 (23.0, 49.0) | 33.0 (24.0, 49.0) | 32.0 (22.8, 53.0) | 0.583 |
| SOFA^h^ (score), Median (IQR) | 4.0 (1.0, 6.0) | 4.0 (1.0, 6.0) | 4.0 (2.0, 6.0) | 0.202 |
| OASIS^i^ (score), Median (IQR) | 22.0 (15.0, 32.0) | 22.0 (15.0, 32.0) | 22.0 (16.0, 31.8) | 0.826 |
| Comorbidities and personal history, n (%) |  |  |  |  |
| Stroke history | 43 ( 7.6) | 38 (8) | 5 (5.6) | 0.42 |
| Long-term anticoagulants | 54 ( 9.6) | 40 (8.4) | 14 (15.6) | 0.035 |
| Hypertension | 183 (32.4) | 147 (31) | 36 (40) | 0.095 |
| Diabetes | 7 ( 1.2) | 3 (0.6) | 4 (4.4) | 0.014 |
| Cerebral edema | 10 ( 1.8) | 8 (1.7) | 2 (2.2) | 0.665 |
| Hydrocephalus | 85 (15.1) | 62 (13.1) | 23 (25.6) | 0.002 |
| Cerebral infarction | 21 ( 3.7) | 17 (3.6) | 4 (4.4) | 0.76 |
| Epilepsy | 40 ( 7.1) | 32 (6.8) | 8 (8.9) | 0.469 |
| Vital signs |  |  |  |  |
| SBP^j^ (mmHg), Median (IQR) | 138.5 (124.2, 155.0) | 139.0 (125.0, 155.0) | 136.0 (121.0, 157.0) | 0.49 |
| DBP^k^ (mmHg), Median (IQR) | 76.0 (66.0, 87.0) | 77.0 (66.2, 87.0) | 74.5 (66.0, 86.2) | 0.56 |
| MBP^l^ (mmHg), Median (IQR) | 92.5 (82.0, 104.0) | 93.0 (82.0, 104.0) | 92.0 (81.8, 101.5) | 0.662 |
| Respiratory rate (breaths/min), Median (IQR) | 18.0 (16.0, 21.0) | 18.0 (16.0, 21.0) | 18.0 (16.0, 21.0) | 0.856 |
| Heart rate (beats/min), Median (IQR) | 81.0 (69.0, 92.0) | 81.0 (70.0, 91.0) | 80.5 (67.0, 96.0) | 0.88 |
| SpO₂ ^m^ (%), Median (IQR) | 98.0 (96.0, 100.0) | 98.0 (96.0, 100.0) | 98.5 (96.0, 100.0) | 0.354 |
| Temperature (°C), Median (IQR) | 36.7 (36.4, 37.1) | 36.7 (36.4, 37.0) | 36.8 (36.4, 37.2) | 0.504 |
| Laboratory parameters |  |  |  |  |
| Hemoglobin (g/dL), Median (IQR) | 13.2 (11.8, 14.5) | 13.3 (11.8, 14.5) | 13.1 (11.6, 14.5) | 0.538 |
| Glucose (mg/dL), Median (IQR) | 142.0 (119.0, 176.0) | 139.0 (118.0, 174.0) | 158.0 (122.0, 188.8) | 0.021 |
| Platelets (109/L), Median (IQR) | 224.0 (185.2, 270.5) | 224.0 (183.0, 269.0) | 230.5 (193.2, 273.0) | 0.383 |
| WBCn (109/L), Median (IQR) | 11.3 (8.7, 14.5) | 11.1 (8.4, 14.0) | 12.7 (10.5, 16.0) | < 0.001 |
| Chloride (mmol/L), Median (IQR) | 104.0 (101.0, 106.0) | 104.0 (101.0, 106.0) | 103.0 (101.0, 106.0) | 0.864 |
| Potassium (mmol/L), Median (IQR) | 3.8 (3.5, 4.2) | 3.8 (3.5, 4.2) | 3.8 (3.5, 4.0) | 0.401 |
| Sodium (mmol/L), Median (IQR) | 138.0 (136.0, 141.0) | 139.0 (136.0, 141.0) | 138.0 (136.0, 141.0) | 0.454 |
| BUN^o^ (mg/dL), Median (IQR) | 14.0 (11.0, 19.0) | 14.0 (10.0, 19.0) | 14.0 (11.0, 18.5) | 0.469 |
| Creatinine (mg/dL), Median (IQR) | 0.8 (0.6, 1.0) | 0.8 (0.6, 1.0) | 0.8 (0.6, 1.0) | 0.312 |
| Bicarbonate (mmol/L) Median (IQR) | 24.0 (22.0, 26.0) | 24.0 (22.0, 26.0) | 24.0 (22.0, 25.8) | 0.18 |
| APTT^p^ (s), Median (IQR) | 27.1 (25.0, 30.0) | 27.4 (25.4, 30.0) | 26.1 (23.9, 29.4) | 0.007 |
| PT (s)^q^, Median (IQR) | 13.2 (12.0, 14.0) | 13.3 (12.5, 14.1) | 11.7 (10.8, 13.6) | < 0.001 |
| INR^r^, Median (IQR) | 1.0 (1.0, 1.1) | 1.0 (1.0, 1.1) | 1.0 (1.0, 1.1) | 0.601 |
| Interventions within 72 h of admission |  |  |  |  |
| Nimodipine | 174 (30.9) | 132 (27.8) | 42 (46.7) | < 0.001 |
| Mechanical ventilation | 217 (38.5) | 178 (37.6) | 39 (43.3) | 0.301 |
| Vasopressors | 71 (12.6) | 47 (9.9) | 24 (26.7) | < 0.001 |
| Endovascular intervention | 126 (22.3) | 100 (21.1) | 26 (28.9) | 0.104 |
| Open surgery | 15 ( 2.7) | 12 (2.5) | 3 (3.3) | 0.718 |
| Clinical Outcomes |  |  |  |  |
| APTT > 87.5 | 13 ( 2.3) | 10 (2.1) | 3 (3.3) | 0.446 |
| Loss hospital | 12.8 (7.5, 19.0) | 12.1 (6.9, 18.1) | 14.9 (12.2, 22.1) | < 0.001 |
| Death in hosp | 84 (14.9) | 75 (15.8) | 9 (10) | 0.155 |

a: SD, standard deviation; b: IQR, interquartile range; c: LOS, length of stay; d: GCS, glasgow coma scale; e: PAASH, prognosis on admission of aneurysmal subarachnoid hemorrhage; f: CCI, charlson comorbidity index; g: SAPS II, simplified acute physiology score II; h: APS III, acute physiology score III; i: SOFA, sequential organ failure assessment; j: OASIS, oxford acute severity of illness score; k: SBP, systolic blood pressure; l: DBP, diastolic blood pressure; m: MBP, mean blood pressure; n: SpO₂, percutaneous oxygen saturation; o: WBC, white blood cell; p: BUN, blood urea nitrogen; q: APTT, activated partial thromboplastin time; r: PT, prothrombin time; s: INR, international normalized ratio; t: N, number.

**Table S4.** Missing values of included individuals.

| Variables | MIMIC-IV cohort | | eICU-CRD cohort | |
| --- | --- | --- | --- | --- |
|  | Missing frequency | Missing percentage% | Missing frequency | Missing percentage |
| INR^a^ | 48 | 7.5829 | 150 | 26.6 |
| APTT^b^ | 48 | 7.5829 | 114 | 20.21 |
| Platelets | 41 | 6.4771 | 199 | 35.28 |
| WBC^c^ | 17 | 2.6856 | 30 | 5.32 |
| Bicarbonate | 17 | 2.6856 | 27 | 4.79 |
| Hemoglobin | 16 | 2.5276 | 36 | 6.38 |
| BUN^d^ | 15 | 2.3697 | 30 | 5.32 |
| Chloride | 14 | 2.2117 | 15 | 2.66 |
| Creatinine | 14 | 2.2117 | 17 | 3.01 |
| Potassium | 14 | 2.2117 | 16 | 2.84 |
| Sodium | 14 | 2.2117 | 14 | 2.48 |
| OASIS^e^ | 13 | 2.0537 | 16 | 2.84 |
| Glucose | 7 | 1.1058 | 0 | 0 |
| SAPSII^f^ | 6 | 0.9479 | 11 | 1.95 |
| GCS^g^ | 6 | 0.9479 | 0 | 0 |
| PAASH^h^ | 3 | 0.4739 | 99 | 17.55 |
| Respiratory rate | 3 | 0.4739 | 99 | 17.55 |
| Temperature | 3 | 0.4739 | 54 | 9.57 |
| DBP^i^ | 3 | 0.4739 | 17 | 3.01 |
| Heart rate | 2 | 0.316 | 34 | 6.03 |
| MBP^j^ | 2 | 0.316 | 24 | 4.26 |
| SBP^k^ | 2 | 0.316 | 34 | 6.03 |
| SPO2^l^ | 2 | 0.316 | 34 | 6.03 |
| SOFA^m^ | 2 | 0.316 | 124 | 21.99 |
| PT^n^ | 1 | 0.158 | 0 | 0 |

a: INR, International Normalized Ratio; b: APTT, Activated Partial Thromboplastin Time; c: WBC, White Blood Cell; d: BUN, Blood Urea Nitrogen; e: OASIS, Oxford Acute Severity of Illness Score; f: SAPS Ⅱ, Simplified Acute Physiology Score Ⅱ; g: GCS, Glasgow Coma Scale; h: PAASH, Prognosis on Admission of Aneurysmal Subarachnoid Hemorrhage; i: DBP, Diastolic Blood Pressure; j: MBP, Mean Blood Pressure; k: SBP, Systolic Blood Pressure; l: SpO₂, Percutaneous Oxygen Saturation; m: SOFA, Sequential Organ Failure Assessment; n: PT, Prothrombin Time

**Table S5.** Sample size and power calculation for the association between heparin use and in-hospital mortality in the MIMIC-IV cohort

| Parameter | Value | Description |
| --- | --- | --- |
| Study groups | Heparin (treatment) vs Non-heparin (control) | Groups compared |
| Outcome | In-hospital death | Primary endpoint |
| Total sample size (N) | 633 | Required total number of subjects |
| Sample size (N1/N2) | 291 / 342 | Control vs. treatment |
| Proportion in control group (%N1) | 45.97% | Percentage allocated to control |
| Number of events (E) | 89 | Total number of expected events |
| Events in groups (E1/E2) | 54.1 / 34.9 | Expected events in control vs. treatment |
| Hazard ratio | 0.58 | Assumed hazard ratio (treatment vs. control) |
| Event probability (Pev1/Pev2) | 0.186 / 0.102 | Probability of event in control vs. treatment |
| Power | 0.820 | Probability of rejecting a false H₀ |
| Alpha | 0.05 | Two-sided significance level |

**Table S6.** Sample size and power calculation for the association between heparin use and in-hospital mortality in the eICU-CRD cohort

| Parameter | Value | Description |
| --- | --- | --- |
| Study groups | Heparin (treatment) vs Non-heparin (control) | Groups compared |
| Outcome | In-hospital death | Primary endpoint |
| Total sample size (N) | 564 | Required total number of subjects |
| Sample size (N1/N2) | 474 / 90 | Control vs. treatment |
| Proportion in control group (%N1) | 84.0% | Percentage allocated to control |
| Number of events (E) | 83.9 | Total number of expected events |
| Events in groups (E1/E2) | 74.9 / 9 | Expected events in control vs. treatment |
| Hazard ratio (HR) | 0.48 | Assumed hazard ratio (treatment vs. control) |
| Event probability (Pev1/Pev2) | 0.158 / 0.1 | Probability of event in control vs. treatment |
| Power | 0.793 | Probability of rejecting a false H₀ |
| Alpha | 0.05 | Two-sided significance level |

**Table S7.** Cox proportional hazards regression model results for heparin cumulative dose in the first 72 hours and mortality outcomes in the MIMIC-IV cohort (adjusted for other models).​

| Outcome | Model | Variable | Dose level | HR^e^ (95% CI^f^) | *P* value |
| --- | --- | --- | --- | --- | --- |
|  |  |  | 0 | 1 (reference) |  |
| In-hospital death | Univariate^a^ | Early heparin total dose | 5000-10000 units | 0.82 (0.49–1.39) | 0.465 |
|  |  |  | 15000units | 0.42 (0.20–0.90) | 0.026 |
|  |  |  | ≥ 20000units | 0.45 (0.21–0.95) | 0.036 |
|  |  |  | P for trend |  | 0.006 |
|  | Model 1^b^ | Early heparin total dose | 5000-10000 units | 0.79 (0.47–1.33) | 0.377 |
|  |  |  | 15000units | 0.41 (0.20–0.88) | 0.021 |
|  |  |  | ≥ 20000units | 0.45 (0.21–0.96) | 0.039 |
|  |  |  | P for trend |  | 0.006 |
|  | Model 2^c^ | Early heparin total dose | 5000-10000 units | 0.76 (0.44–1.31) | 0.318 |
|  |  |  | 15000units | 0.44 (0.21–0.96) | 0.039 |
|  |  |  | ≥ 20000units | 0.53 (0.24–1.15) | 0.106 |
|  |  |  | P for trend |  | 0.006 |
|  | Model 3^d^ | Early heparin total dose | 5000-10000 units | 0.93 (0.53–1.62) | 0.798 |
|  |  |  | 15000units | 0.43 (0.20–0.94) | 0.035 |
|  |  |  | ≥ 20000units | 0.48 (0.21–1.08) | 0.075 |
|  |  |  | P for trend |  | 0.006 |
| Death within 28 days | Univariate | Early heparin total dose | 5000-10000 units | 0.67 (0.40–1.12) | 0.125 |
|  |  |  | 15000units | 0.53 (0.29–0.99) | 0.047 |
|  |  |  | ≥ 20000units | 0.32 (0.15–0.71) | 0.005 |
|  |  |  | P for trend |  | <0.001 |
|  | Model1 | Early heparin total dose | 5000-10000 units | 0.63 (0.38–1.06) | 0.083 |
|  |  |  | 15000units | 0.52 (0.28–0.98) | 0.042 |
|  |  |  | ≥ 20000units | 0.32 (0.15–0.70) | 0.004 |
|  |  |  | P for trend |  | <0.001 |
|  | Model2 | Early heparin total dose | 5000-10000 units | 0.62 (0.36–1.06) | 0.082 |
|  |  |  | 15000units | 0.58 (0.31–1.10) | 0.094 |
|  |  |  | ≥ 20000units | 0.42 (0.19–0.92) | 0.031 |
|  |  |  | P for trend |  | <0.001 |
|  | Model3 | Early heparin total dose | 5000-10000 units | 0.71 (0.42–1.23) | 0.222 |
|  |  |  | 15000units | 0.57 (0.30–1.09) | 0.088 |
|  |  |  | ≥ 20000units | 0.40 (0.18–0.90) | 0.027 |
|  |  |  | P for trend |  | <0.001 |
| Death within 90 days | Univariate | Early heparin total dose | 5000-10000 units | 0.67 (0.42–1.06) | 0.09 |
|  |  |  | 15000units | 0.64 (0.38–1.07) | 0.087 |
|  |  |  | ≥ 20000units | 0.40 (0.21–0.76) | 0.005 |
|  |  |  | P for trend |  | 0.002 |
|  | Model1 | Early heparin total dose | 5000-10000 units | 0.64 (0.40–1.01) | 0.057 |
|  |  |  | 15000units | 0.62 (0.37–1.05) | 0.074 |
|  |  |  | ≥ 20000units | 0.40 (0.21–0.75) | 0.004 |
|  |  |  | P for trend |  | 0.002 |
|  | Model2 | Early heparin total dose | 5000-10000 units | 0.61 (0.38–0.98) | 0.041 |
|  |  |  | 15000units | 0.71 (0.42–1.21) | 0.213 |
|  |  |  | ≥ 20000units | 0.49 (0.26–0.93) | 0.029 |
|  |  |  | P for trend |  | 0.002 |
|  | Model3 | Early heparin total dose | 5000-10000 units | 0.72 (0.45–1.17) | 0.186 |
|  |  |  | 15000units | 0.70 (0.41–1.20) | 0.196 |
|  |  |  | ≥ 20000units | 0.47 (0.24–0.91) | 0.025 |
|  |  |  | P for trend |  | 0.002 |
| Death within 180 days | Univariate | Early heparin total dose | 5000-10000 units | 0.69 (0.44–1.06) | 0.091 |
|  |  |  | 15000units | 0.65 (0.40–1.05) | 0.08 |
|  |  |  | ≥ 20000units | 0.40 (0.22–0.73) | 0.003 |
|  |  |  | P for trend |  | <0.001 |
|  | Model1 | Early heparin total dose | 5000-10000 units | 0.65 (0.42–1.01) | 0.055 |
|  |  |  | 15000units | 0.63 (0.39–1.03) | 0.068 |
|  |  |  | ≥ 20000units | 0.39 (0.21–0.72) | 0.003 |
|  |  |  | P for trend |  | <0.001 |
|  | Model2 | Early heparin total dose | 5000-10000 units | 0.63 (0.40–0.99) | 0.046 |
|  |  |  | 15000units | 0.73 (0.44–1.21) | 0.221 |
|  |  |  | ≥ 20000units | 0.49 (0.27–0.92) | 0.025 |
|  |  |  | P for trend |  | <0.001 |
|  | Model3 | Early heparin total dose | 5000-10000 units | 0.72 (0.45–1.13) | 0.149 |
|  |  |  | 15000units | 0.72 (0.43–1.19) | 0.199 |
|  |  |  | ≥ 20000units | 0.46 (0.24–0.87) | 0.017 |
|  |  |  | P for trend |  | <0.001 |
| Death within 365 days | Univariate | Early heparin total dose | 5000-10000 units | 0.67 (0.44–1.02) | 0.06 |
|  |  |  | 15000units | 0.61 (0.38–0.99) | 0.045 |
|  |  |  | ≥ 20000units | 0.42 (0.24–0.73) | 0.002 |
|  |  |  | P for trend |  | <0.001 |
|  | Model1 | Early heparin total dose | 5000-10000 units | 0.63 (0.41–0.96) | 0.031 |
|  |  |  | 15000units | 0.60 (0.38–0.97) | 0.039 |
|  |  |  | ≥ 20000units | 0.41 (0.23–0.73) | 0.002 |
|  |  |  | P for trend |  | <0.001 |
|  | Model2 | Early heparin total dose | 5000-10000 units | 0.62 (0.40–0.96) | 0.03 |
|  |  |  | 15000units | 0.70 (0.43–1.14) | 0.15 |
|  |  |  | ≥ 20000units | 0.51 (0.29–0.90) | 0.02 |
|  |  |  | P for trend |  | <0.001 |
|  | Model3 | Early heparin total dose | 5000-10000 units | 0.68 (0.44–1.05) | 0.08 |
|  |  |  | 15000units | 0.68 (0.42–1.12) | 0.128 |
|  |  |  | ≥ 20000units | 0.48 (0.27–0.86) | 0.014 |
|  |  |  | P for trend |  | <0.001 |

a: No covariates adjusted.

b: Model 1, Adjusted for age, gender, and race.

c: Model 2, Adjusted for age, gender, race, GCS, PAASH, SAPSII, APSIII, OASIS, CCI, SOFA.

d: Model 3, Adjusted for variables with P < 0.05 in Table 1, including GCS, PAASH, SAPSII, APSIII, OASIS, glucose, WBC, BUN, hydrocephalus, mechanical ventilation, nimodipine, and open surgery.

e: hazard ratio

f: confidence interval
